# Genitourinary Defects Associated with Genomic Deletions in 2p15 Encompassing *OTX1*

**DOI:** 10.1371/journal.pone.0107028

**Published:** 2014-09-09

**Authors:** Carolina J. Jorgez, Jill A. Rosenfeld, Nathan R. Wilken, Hima V. Vangapandu, Aysegul Sahin, Dung Pham, Claudia M. B. Carvalho, Anne Bandholz, Amanda Miller, David D. Weaver, Barbara Burton, Deepti Babu, John S. Bamforth, Timothy Wilks, Daniel P. Flynn, Elizabeth Roeder, Ankita Patel, Sau W. Cheung, James R. Lupski, Dolores J. Lamb

**Affiliations:** 1 Center for Reproductive Medicine, Baylor College of Medicine, Houston, Texas, United States of America; 2 Scott Department of Urology, Baylor College of Medicine, Houston, Texas, United States of America; 3 Department of Molecular and Cellular Biology, Baylor College of Medicine, Houston, Texas, United States of America; 4 Department of Molecular and Human Genetics, Baylor College of Medicine, Houston, Texas, United States of America; 5 Department of Pediatrics, Baylor College of Medicine, Houston, Texas, United States of America; 6 Signature Genomic Laboratories, PerkinElmer, Inc., Spokane, Washington, United States of America; 7 Department of Medical and Molecular Genetics, Indiana University School of Medicine, Indianapolis, Indiana, United States of America; 8 Ann & Robert H. Lurie Children's Hospital of Chicago, Chicago, Illinois, United States of America; 9 University of Alberta, Edmonton, Alberta, Canada; 10 Madigan Army Medical Center, Department of Pediatrics, Tacoma, Washington, United States of America; 11 Department of Children's Endocrinology, St. Luke's Children's Specialty Center, Boise, Idaho, United States of America; 12 Department of Pediatrics, University of Texas Health Science Center at San Antonio, San Antonio, Texas, United States of America; Centro Nacional de Investigaciones Oncológicas (CNIO), Spain

## Abstract

Normal development of the genitourinary (GU) tract is a complex process that frequently goes awry. In male children the most frequent congenital GU anomalies are cryptorchidism (1–4%), hypospadias (1%) and micropenis (0.35%). Bladder exstrophy and epispadias complex (BEEC) (1∶47000) occurs less frequently but significantly impacts patients' lives. Array comparative genomic hybridization (aCGH) identified seven individuals with overlapping deletions in the 2p15 region (66.0 kb-5.6 Mb). Six of these patients have GU defects, while the remaining patient has no GU defect. These deletions encompass the transcription factor *OTX1*. Subjects 2–7 had large *de novo* CNVs (2.39–6.31 Mb) and exhibited features similar to those associated with the 2p15p16.1 and 2p15p14 microdeletion syndromes, including developmental delay, short stature, and variable GU defects. Subject-1 with BEEC had the smallest deletion (66 kb), which deleted only one copy of *OTX1*. *Otx1-*null mice have seizures, prepubescent transient growth retardation and gonadal defects. Two subjects have short stature, two have seizures, and six have GU defects, mainly affecting the external genitalia. The presence of GU defects in six patients in our cohort and eight of thirteen patients reported with deletions within 2p14p16.1 (two with deletion of *OTX1*) suggest that genes in 2p15 are important for GU development. Genitalia defects in these patients could result from the effect of *OTX1* on pituitary hormone secretion or on the regulation of SHH signaling, which is crucial for development of the bladder and genitalia.

## Introduction

The genitourinary (GU) tract is a multicomponent organ system in which mesenchymal-epithelial interactions play a critical developmental role [1]. Imbalance of this tightly regulated interaction can cause congenital birth defects. GU defects are among the most common male congenital anomalies and include cryptorchidism (1%–4%) [2], hypospadias (1%) [3], [4], and micropenis (0.35% of full-term newborns) [4]. Vesicoureteral-reflux (VUR) is another common GU defect affecting 1% of newborns [5]. In contrast, bladder exstrophy and epispadias complex (BEEC) occurs in 1∶47,000 newborns [6], [7]. It is a more severe urologic malformation characterized by abnormal invagination of the bladder through the abdominal wall and an abnormal urethral opening.

These GU defects can have long-term sequelae. Cryptorchidism is associated with infertility and testicular cancer [8]–[13]. Azoospermia is reported in men with unilateral cryptorchidism (13%) and untreated bilateral cryptorchidism (89%) [8]. Orchidopexy performed before 2 years of age minimizes germ cell loss, but there is a significant difference in the ability and time required to father a child in men with bilateral cryptorchidism (65.3%). This decline in fertility is not evident in men with unilateral cryptorchidism (89.7%), when compared to controls (93.7%) [14], [15]. Nearly 5–10% of men who develop germ cell tumors have a history of cryptorchidism [11], [13]. BEEC significantly affects patients' lives psychologically, socially, and sexually with patients reporting anxiety and low self-esteem due to abnormalities of the genitalia and erectile and orgasmic dysfunction [16], [17]. In addition, some BEEC patients exhibit elevated FSH and spermatogenic failure [18]. While GU defects frequently occur as isolated defects, they present together with multiple GU defects, usually known as CAKUTs (congenital anomalies of the kidney and urinary tract), that occur in 1∶500 live births [19]. Emerging evidence suggests that genetic and genomic changes can result in susceptibility to abnormal GU tract development [20].

Array-comparative-genomic-hybridization (aCGH) is a powerful genetic tool for detecting copy-number variants (CNVs), identifying new genetic syndromes, and correlating genotype and phenotype abnormalities more precisely than routine karyotyping. Two newly discovered syndromes involve CNV abnormalities within chromosome 2p. The first, chromosome 2p15p16.1 microdeletion syndrome, was reported in ten individuals with deletions ranging from chr2:55.48–63.33 Mb (UCSC-hg18 build of the human genome). These patients have common facial features and intellectual disability, and 70% of them present with GU defects. The GU defects include those involving the testis (4/4), external genitalia (2/10), and kidneys (4/10). The three individuals without GU defects were females [21]–[29]. The second syndrome, chromosome 2p14p15 microdeletion syndrome, was reported in three individuals with deletions ranging from chr2:61.97-65.98 Mb [30], [31]. These patients have mild intellectual disability; one has cryptorchidism.

Located between the two microdeletion syndromes' critical intervals and deleted in three patients identified in clinical databases is the orthodenticle-homolog-1 (*OTX1*) gene (63.13–63.14 Mb). OTX1 is a transcription factor with important roles in controlling specification, maintenance, and regionalization of the vertebrate brain. *Otx1* null mice suffer from spontaneous epilepsy with focal, as well as generalized seizures [32]. Also, they display a transient decrease in gonadal size with profound architectural changes [33].

**Figure 1:**
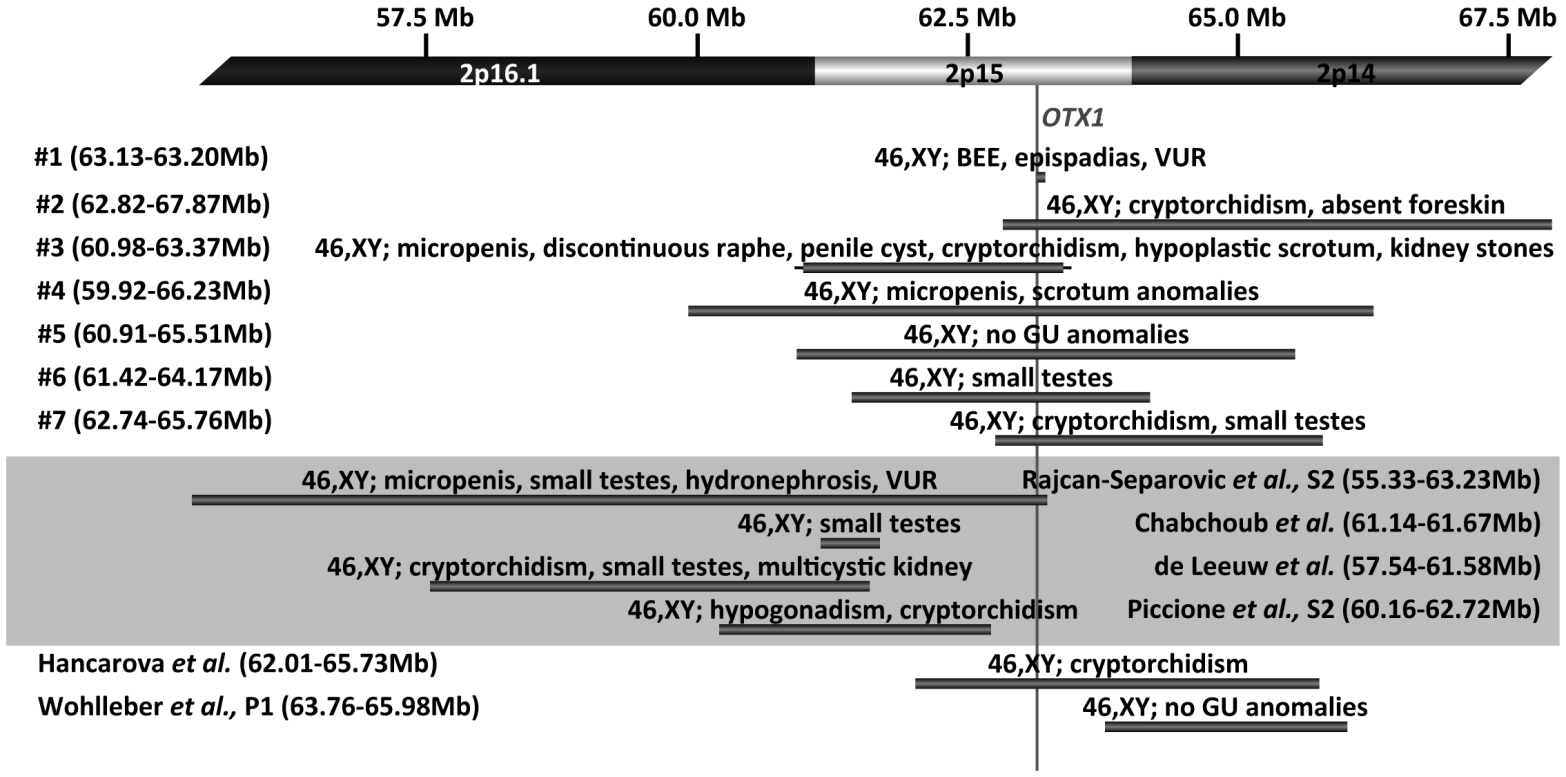
Schematic of the chromosome 2p regions deleted in our subjects and in those males identified in the literature. Top panel represents chromosome 2p16.1p14 showing the seven male subjects in our study together with the size of their microdeletions, as well as a brief description of the of GU defects present in each patient. Gray rectangles represent the minimum deletion regions and horizontal black lines extend through gaps in coverage to show the maximum deletion size. The location of *OTX1* (the only gene commonly deleted in all seven of our subjects) is indicated by a vertical line. Middle and bottom panels show all the male patients described with the 2p15p16.1 and 2p14p15 microdeletion syndromes, respectively, indicating the size of the deletion and their GU defects. Coordinates shown correspond to the hg18 build of the human genome.

This study identified six subjects with GU defects harboring a deletion in 2p15 who share an interval of minimal overlap corresponding with the coding regions of *EHBP1*, *OTX1*, and *WDPC* in all but one subject, whose deletion only involved *OTX1*. The presence of these defects suggests that this region has a role in GU development (Fig. 1).

## Materials and Methods

### Selection of Study Subjects

Three different protocols were used. Two were approved and overseen by the Institutional Review Board of Baylor College of Medicine (BCM) and the other by the Institutional Review Board-Spokane at Signature Genomics (SG). The first protocol from BCM included a cohort of 30 BEEC probands and 85 controls analyzed using a research aCGH from NimbleGen containing 720,000 probes from 2008–2012. The second protocol from BCM covers 18,734 probands tested by clinical aCGH at BCM Medical Genetic Laboratories from 2008–2012. The SG protocol included 30,183 probands tested by clinical aCGH from 2007–2010. The probands referred for clinical aCGH display a range of clinical conditions. Probands were recruited independent of race, ethnicity, and age. Approved informed consents were obtained from the parents or legal guardians. Blood was collected from subjects and controls with normal genitourinary development. Blood or saliva was collected from the subjects' immediate family when available. DNA was extracted using the Qiagen Puregene DNA extraction kit (Valencia, CA) according to the manufacturer's protocol.

### Clinical Reports

**Subject-1** (63.13 Mb-63.20 Mb): A 10-year-old male was the first child of healthy, non-consanguineous parents. Family history showed no significant problems. He was delivered vaginally at 40 weeks following a pregnancy complicated by maternal hypertension. At birth, the patient had BEEC and underwent bladder exstrophy repair followed by epispadias repair. He later developed a urethral cutaneous fistula and bilateral VUR. He underwent bladder neck closure, urethral-cutaneous fistula repair, Mitrofanoff creation, and bilateral ureteral re-implant. The urologist's report indicated that the patient's development appeared grossly normal without dysmorphic features. A formal examination was not performed by a clinical geneticist.

**Subject-2** (62.82 Mb–67.87 Mb) was a 4-year-old male born via vaginal delivery at term to a 36-year-old G7P2 mother. The mother took progesterone for the first 11 weeks of pregnancy. Birth weight was 3.28 kg (25^th^ percentile) and length was 53.3 cm (75^th^–90^th^ percentile). He required supplemental oxygen for several minutes after birth. Genital anomalies included unilateral right cryptorchidism (surgically repaired) and congenital absence of the foreskin. At age 2, he had an adenoidectomy and ear tube placement. He is developmentally delayed and has speech apraxia. At age 4, he uses 3–4 word phrases, and his receptive language is better than his expressive language. He has delayed visual maturation and a history of mild hypotonia. Dysmorphic features include metopic ridging, hooded eyelids, a prominent nose, a thin upper lip, a slightly protruding tongue, and slightly protruding ears. He also has hirsutism on his back and a single pigmented macule on his penis.

**Subject-3** (60.98 Mb–63.37 Mb) is an 11-year-old male born at 37 weeks via Cesarean section to a 20-year-old G1P0 mother following a pregnancy complicated by placenta previa (which resolved) and maternal hypertension beginning at 34 weeks of gestation. Birth weight (2.56 kg) and length (47 cm) were between the 3^rd^ and 10^th^ percentiles. Duodenal web and malrotation were diagnosed neonatally and surgically repaired. He had slow growth during the neonatal period. Endocrine studies were normal. Genital anomalies included a hypoplastic scrotum, unilateral right cryptorchidism (testes were each 1cc), discontinuous prominent raphe, micropenis (phallus measuring 1.8 cm at 4 months, <3^rd^ percentile for his age), and a 1 cm cyst on the penis. Orchidopexy was performed. He had kidney stones. He required a cholecystectomy at age 4. He had moderate to severe developmental delays, intellectual disabilities, vision problems, ptosis, recurrent ear infections, febrile seizures, chronic nosebleeds, mixed hypo/hypertonia, and GI motility problems resulting in vomiting, diarrhea, and constipation. Dysmorphic features include a flattened occiput, persistence of hair on the lateral forehead, metopic ridging, prominent superior orbital ridges, telecanthus, reverse epicanthal folds, long eyelashes, downslanting palpebral fissures, hypoplastic alae nasi with bulbous tip, a narrow palate, a prominent upper lip, micrognathia, and prominent ears with underdeveloped helices. He has pes planus, mild finger tapering, tightness of the knees, hypoplastic pectoralis major, and hypotrophic lower leg muscles. His growth measurements at 9 years, 5 months were height 115.5 cm (3^rd^ percentile), weight 19.3 kg (3^rd^ percentile), and OFC 47.6 cm (-2 SD). At age 10, he started having seizures, which are controlled with medications. At age 11, he attended special education and regular classes without behavioral problems.

**Subject-4** (59.92 Mb-66.23 Mb) is a 21-month-old male delivered vaginally at 39+6 weeks to a 24-year-old G4P2SAb1 mother with a history of uterine prolapse. Prenatal ultrasound was remarkable for “stomach debris”, which resolved on subsequent ultrasounds. His birth weight was 2.98 kg (25^th^–50^th^ percentile) and length 47.3 cm (50^th^ percentile). At birth he had hypotonia and dysmorphic features including a very small anterior fontanelle with ridged sutures, a beaked prominent nose with short columella, micropenis with bilateral testes palpable in a small scrotum, and rocker-bottom feet. His karyotype was normal. He had small, downslanting palpebral fissures with epicanthal folds, sparse eyebrows, and bilateral elbow dimples. He had microcephaly and poor growth. His brain MRI revealed diffuse cerebral atrophy, prominent ventricles suggestive of colpocephaly, and an enlarged cisterna magna. Further workup revealed mesocardia and right-sided cross-fused renal ectopia. At 17 months he had motor skills in the 6–8 month range and language/social skills in the 9–10 month range. His failure to thrive and global developmental delay were complicated by frequent upper respiratory infections and gastroesophageal reflux.

**Subject-5** (60.91 Mb–65.51 Mb) is a 16-year-old male born at term with a birth weight of 3.01 kg (10^th^–25^th^ percentile). At birth microcephaly was present and his face was asymmetrical. As an infant, he had periods of airway obstruction, cyanosis, feeding difficulties with recurrent aspiration pneumonias, and failure to thrive. Developmentally he was delayed, walking after 2 years. He had a disturbed sleeping pattern as a child, which improved with time. He had difficulties with short attention span and aggression. He suffered a single seizure following a motor vehicle accident. At present, he has osteopenia, detected from radiographs following a fall. At age 16, height and weight are within normal limits. Dysmorphic features include brachycephaly, a high and prominent nasal bridge, left ptosis, synophrys, malar hypoplasia, a large mouth, a prominent lower lip, a notch over his left earlobe and a flattened right occiput. He had mild mid-thoracic scoliosis, barrel chest with low placed nipples, bilateral single palmar creases, slender hands and feet with long fingers and toes, pes planus with prominent heels, and an eversion of the left ankle. Reflexes in the lower limbs were brisk. He has no known GU defects but has not had a renal ultrasound.

**Subject-6** (61.42 Mb–64.17 Mb) is a 14-year-old male born after induction at 38 weeks. Pregnancy was complicated by severe nausea gravidarum. His birth weight was 2.84 kg (10^th^–25^th^ percentile) with a length of 49.5 cm (50^th^–75^th^ percentile). Apgar scores were 7 and 8 at 1 and 5 minutes, respectively. In the first week he had jaundice that spontaneously resolved. He was referred to a pediatric endocrinology clinic at age 14 to evaluate short stature (height 146 cm, (3^rd^ percentile) and weight 31.9 kg (1^st^ percentile)). He had pervasive developmental and speech delay. He has a history of chronic and significant sinusitis and generalized hypotonia. Dysmorphic features include a small bitemporal diameter, dolichocephaly with a prominent forehead, epicanthal folds, long eyelashes, hypertelorism, right otosclerosis, and a very high-arched mouth palate. He has some degree of clinodactyly. The chest wall is asymmetric. He has small testes (5 ml), but his penile length and girth are considerably larger than expected with his current development. He has a history of leukopenia and possible cyclic neutropenia. He has had significant infections, especially involving his teeth over the years. He had 2 cranial surgeries, a cranial distraction at 6 months and a cranial vault revision at age 4 due to metopic sutures.

**Subject-7** (62.74 Mb–65.76 Mb) is a 4-year-old male born at term to a 24-year-old G2P1-2 mother. His birth weight was 3.29 kg (25^th^ percentile). At 30 months the child had developmental delay and dysmorphic features. He rolled at 8–9 months, sat at 12 months, walked at 18 months, and spoke at 26 months. His growth has been normal, with weight in the 90^th^ percentile. Facial features include right ptosis, short palpebral fissures (<5^th^ percentile), large ears (>97^th^ percentile), a long nose, a smooth and somewhat long philtrum, and a thin upper lip. He also has a left Sydney crease, mild right third and fourth finger camptodactyly, pes planus, prominent heels, and bilateral esotropia. Behavior is abnormal with significant hyperactivity. At 30 months the left testicle was nonpalpable in the scrotum or inguinal canal and the right testicular volume seemed small for age. At age 4 he is essentially nonverbal.

### aCGH and Validation

Subject-1 as well as 29 subjects with BEEC and 85 controls without GU defects were analyzed using the NimbleGen 3×720K aCGH (Madison, WI). Controls were sex matched to subjects. The NimbleGen aCGH samples were processed at the Roche Service Lab (Iceland). Data were analyzed using Nexus-Copy-Number (BioDiscovery) and SignalMap (Roche). To validate the aCGH, TaqMan-CNV assays were run for *OTX1* (Hs02720160_cn). TaqMan CNV reactions were performed in triplicate as previously described [34]. To determine the size, genomic extent, and gene content for subject-1's rearrangement, we designed a tiling-path aCGH spanning the 2p15 chromosomal region. A custom Agilent aCGH 8×60 K (Santa Clara, CA) was designed using the Agilent e-array website (http://earray.chem.agilent.com/earray/). We selected 7,500 probes covering chr2:62,353,504–63,853,504, which represents an average distribution of one probe every 200 bp. Probe labeling and hybridization were performed as described [35]. Subject-1's father was unavailable for testing; the mother was tested for *OTX1* CNVs using TaqMan-CNV assays and long range PCR.

DNA from subjects-2, -4, and -5 was analyzed using a 135K, whole-genome, oligonucleotide-based array (SignatureChipOS v2.0 [subjects 2 & 5] or v3.0 [subject 4], custom-designed by SG, Spokane, WA, manufactured by NimbleGen) using previously described methods [36]. DNA from subject-3 was analyzed using a 105K, whole-genome, oligonucleotide-based array (SignatureChipOS v1.0, custom-designed by SG, manufactured by Agilent) according to previously described methods [37]. Deletions in the probands were confirmed by fluorescence *in situ* hybridization (FISH) with BAC clones from deleted regions using previously published methods [38]. Dual color FISH analysis to visualize the deletion and test the parents was performed using clones RP11-1073G3 (subject-2; chr2:63066431-63258232-hg18), RP11-477N2 (subject-3; chr2:61,395,080-61,588,096-hg18), RP11-139C22 (subject-4; chr2:60605798-60780422-hg18) and RP11-120J10 (subject-5; chr2:61193500-61369219-hg18). Either RP11-477N2 (chr2:61,395,080-61,588,096-hg18) or D2Z2 (chr2 centromere) were used as a control.

DNA from subjects-6 and -7 was analyzed using a 180K custom-designed, exon targeted (∼1700 genes), whole genome oligonucleotide-based array (BCM V.8 and/or V8.1 manufactured by Agilent) and confirmed using FISH as described previously [39]. Dual color FISH analysis was performed in subjects using clones RP11-451M22 (chr2:64676181-64860244-hg18) and RP11-681L4 (chr2:61937334-61937694-hg18) with CEN2 (chr2 centromere) as the control. Parents of subject-6 were studied by FISH analysis. Parents of subject-7 were tested by BCM-V8 array because subject-7 had three CNVs: a 2p15 microdeletion and two small microduplications below the resolution for FISH analysis.

Clinical aCGH testing was performed from 2007–2012 and analyzed immediately. All subjects tested by aCGH at BCM and SG with CNVs including *OTX1* and available clinical data were included in this study.

### *OTX1* Sequence

For PCR reactions, 50 ng of gDNA were amplified using Phusion High-Fidelity PCR (NEB). After PCR, the product was purified using the ExoSAP-It kit (USB Scientific, Cleveland, OH). Purified products were sequenced using Sanger sequencing combined with ABI 3730*xl* DNA analyzers for capillary electrophoresis and fluorescent dye terminator detection (Genewiz). Data were analyzed using Surveyor software (Softgenetics).

### Long-range PCR Amplification

Long-range PCR was performed using TaKaRa-LA-Taq (TAKARA-Bio). A 25 µl PCR reaction was performed using 1.25U TaKaRa enzyme, 0.4 mM dNTP, 0.2 µM of primer forward (ccttgacttgccctcacact) and reverse (gcctaatcccctttgcctta), 1 M betaine and 250 ng of DNA template. The PCR conditions were as follows: 98°C (30 s); then 32 cycles of 94°C (60 s), 65°C (20 s) and 68°C (20 m); finally 68°C (10 m). One fraction of the amplification product was electrophoresed on a 1% agarose gel. The other fraction was purified using the ExoSAP-It kit (USB Scientific, Cleveland, OH). Purified products were sequenced using Sanger sequencing at Genewiz. Data were analyzed using bioinformatics databases http://blast.ncbi.nlm.nih.gov and http://genome.ucsc.edu.

## Results

### Identification of CNVs in 2p15 in Subjects with GU defects

**Figure 2:**
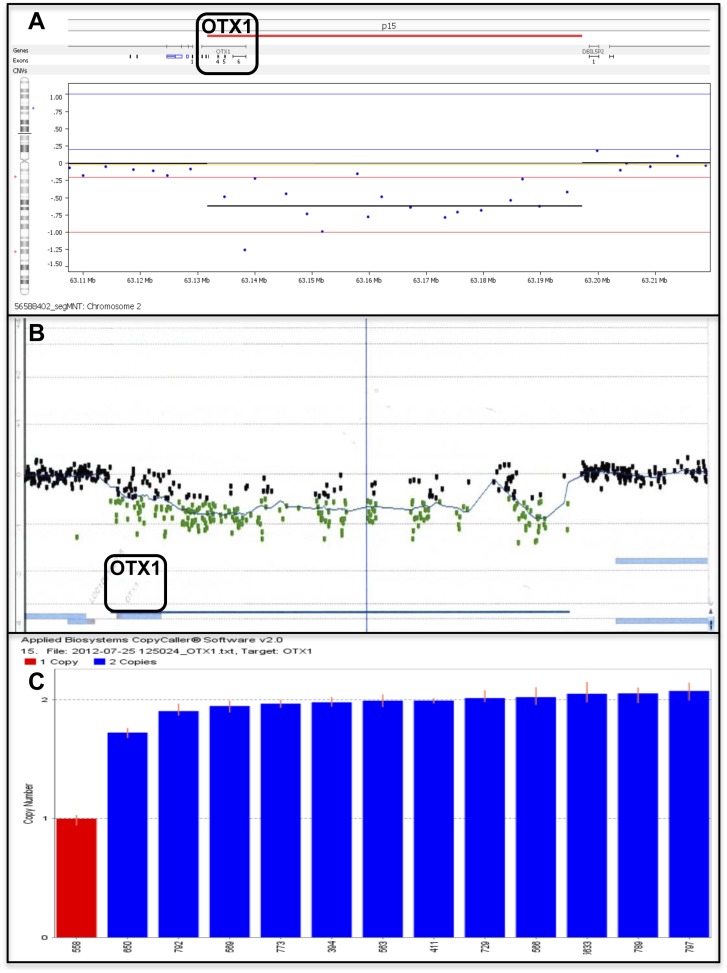
Analysis of an *OTX1* deletion in subject 1 with BEEC. A) NimbleGen aCGH revealed a microdeletion of 66 kb at 2p15 that encompasses only *OTX1* (blue dots indicate probe position). B) Validation of NimbleGen aCGH results with a custom Agilent aCGH for that microdeletion. Green dots indicate the area of microdeletion. C) *OTX1*-Taqman-CNV assays validated the microdeletion showing one copy of the gene (red bar) in the subject-1 and 12 other BEEC subjects with normal copy number (blue bars).

**Figure 3:**
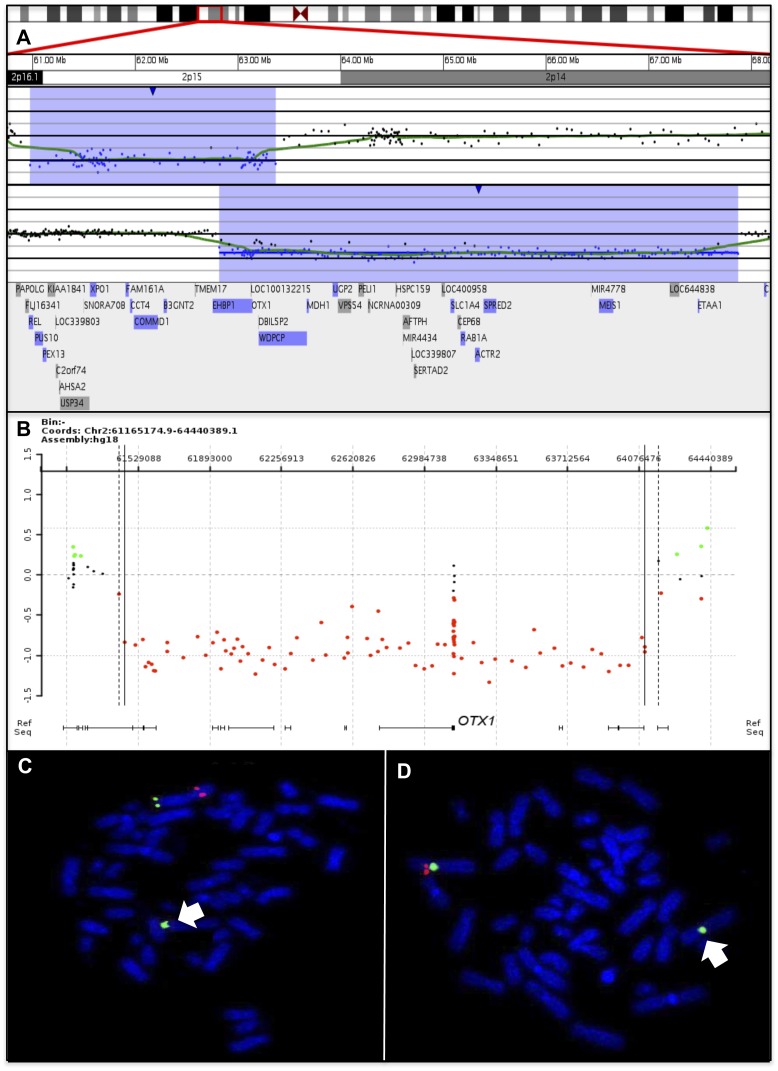
Clinical diagnostic arrays identify subjects with microdeletions in 2p15. A) Signature Genomics array analysis revealed a microdeletion in 2p15 that encompasses *OTX1* (red line) in subject-2 and -3. B) Baylor Clinical Genetics array revealed a microdeletion (red dots) in 2p15 that encompasses *OTX1* in subject-6 (red box). Coordinates shown are according to the hg18 build of the human genome. C) Dual color FISH analysis using spectrum red labeled clone RP11-1073G3 and spectrum green labeled clone RP11-367H1 revealed the deletion of 2p15 on one homolog (arrow) in subject-2. D) Dual color FISH analysis using spectrum red labeled clone RP11-477N2 and spectrum green labeled clone D2Z2 revealed the deletion of 2p15 on one homolog (arrow) in subject-3.

We identified seven subjects with deletions of the 2p15 region ranging from 66 kb to 6.3 Mb in size (Fig. 1). Four subjects' deletions extend distally, at least partially overlapping the proposed critical region for the 2p15p16.1 microdeletion syndrome, and five subjects' deletions extend proximally into 2p14. Three subjects' deletions extend in both directions and only subject-1 (smallest deletion) lacks these other regions. Subject-1 was identified in a cohort of 30 BEEC patients using NimbleGen 3×720 aCGH (Fig. 2A). His deletion was validated using a custom aCGH from Agilent (Fig. 2B) and qPCR CNV-Taqman assays (Fig. 2C). None of the remaining 29 BEEC subjects or the 85 controls without GU defects have CNVs in *OTX1*. Subjects 2–5 were identified among 30,183 probands tested by aCGH at SG (Fig. 3A and data not shown) and validated by FISH (Fig. 3C–D and data not shown). Two additional subjects with *OTX1* deletions were identified at SG, but their clinical information was unavailable for publication. FISH testing on the parents of subjects 3–5 showed the children's deletions to be *de novo*. Subjects 6–7 were detected from 18,734 probands tested by aCGH at BCM Medical Genetic Laboratories (Fig. 3B and data not shown) and validated by FISH (data not shown). Parental testing for subjects 6–7 showed the children's deletions to be *de novo*. The common region deleted in these subjects included only *OTX1* at 2p15 (Fig. 1). No additional CNVs of known or suspected clinical significance were detected in these subjects. Subject-7 had three CNVs: a 2p15 microdeletion and two small microduplications. The two microduplications were maternaly inherited and of unclear significance, one at 17q24.2 (62550505-62819345 encompassing the *HELZ, PSMD12* and *PITPNC1* genes) and another at Xp22.31 (7980296-8075153 that involves only *MIR651*).

Among these individuals, clinical features varied with some phenotypic overlap with the currently described 2p15p16.1 and 2p14p15 microdeletion syndromes. However, all subjects with the exception of subject-5 are reported to have GU defects (Table 1) that mainly involve the genitalia (testes (4/7), penis (3/7) and scrotum (2/7)). Five subjects had multiple GU defects (Table 1).

**Table 1:** Comparison of the clinical features of male patients with deletion in 2p14-p16.1 that encompasses *OTX1*.

Subjects	1	2	3	4	5	6	7	Rajcan et al.	Hancarova et al.
Size of Deletion (Mb)	0.07	5.05	2.39	6.31	4.59	2.75	3.02	7.89	3.72
Chromosome Localization	2p15	2p15p14	2p16.1p15	2p16.1p14	2p16.1p14	2p15p14	2p15p14	2p16.1p15	2p15p14
Genomic Region (Mb, hg18)	63.13–63.20	62.82–67.87	60.98–63.37	59.92–66.23	60.91–65.51	61.42–64.17	62.74–65.76	55.33–63.23	62.01–65.73
**General**									
Age at Evaluation (years)	10	4	9	1.75	16	14	4	7	4
Sex	Male	Male	Male	Male	Male	Male	Male	Male	Male
Developmental Delay	-	+	+	+	+	+	+	+	+
Seizures	-	-	+	-	+	-	-	-	-
Feeding Problems	-	-	+	+	+	-	-	+	+
Vision Problems	-	+	+	-	-	-	+	+	+
Recurrent Ear Infections	-	+	+	-	-	+	-	-	-
Short Stature	-	-	+	-	-	+	-	+	-
Microcephaly	-	-	-	+	+	-	-	+	+
Hypotonia	-	+	+	+	-	+	-	-	+
**Urological Features**									
Bladder Exstrophy	+	-	-	-	-	-	-	-	-
Epispadias	+	-	-	-	-	-	-	-	-
VUR	+	-	-	-	-	-	-	+	-
Cryptorchidism	-	+	+	-	-	-	+	-	+
Small Testes	-	-	+	-	-	+	+	+	-
Absent Foreskin	-	+	-	-	-	-	-	-	-
Scrotal anomalies	-	-	+	+	-	-	-	-	-
Micropenis	-	-	+	+	-	-	-	+	-
Kidney Abnormalities	-	-	+	-	-	-	-	+	-
**Facial Features**									
Flattened Occiput	NM	-	+	-	+	-	NM	+	+
Metopic Ridging	NM	+	+	NM	NM	-	NM	+	-
Ptosis	NM	-	+	-	+	-	+	+	-
Slanted Palpebral Fissures	NM	-	+	+	NM	-	+	+	-
Epicanthal Folds	NM	+	+	+	NM	+	NM	+	-
Prominent Nose	NM	+	+	+	+	-	+	+	+
Long, Straight Eyelashes	NM	-	+	NM	NM	+	NM	+	-
Large Ears	NM	+	+	NM	-	-	+	+	+
Thin Upper Lip	NM	+	-	-	-	-	+	-	+
**Other**									
Nipple Abnormalities	NM	-	-	NM	+	-	-	+	-
Pes Planus	NM	-	+	-	+	-	+	-	-
Attention Deficit Behavior	NM	-	-	NM	+	+	NM	+	+

VUR  =  vesicoureteral reflux; NM  =  not mentioned.

### Analysis of Exon Sequence and Deletion Breakpoints

To assess whether *OTX1* was the only gene deleted in subject-1, long-range PCR was used to define the breakpoints of the deletion. Subject-1's deletion was located at chr2:63,130,672–63,196,654 (data not shown), a region that only encompasses *OTX1*. There are two *OTX1* variants that encode the same protein but differ in the 5′UTR, NM_014562.3 (chr2:63,131,441–130,696–63,138,470) and NM_001199770.1 (chr2:63,130,696–63,138,470), and both variants are deleted. The two genes flanking *OTX1*, EH domain binding protein 1 (*EHBP1*) (chr2:62,754,490–63,127,125) and WD repeat containing planar cell polarity effector (*WDPCP*) (chr2:63,202,039–63,669,371), were not included in the deleted region. Long range PCR analysis of the mother's DNA did not show the deletion and the father's DNA was unavailable for testing; consequently, inheritance could not be established.

In addition, we performed Sanger sequencing of *OTX1* on subject-1 as well as 29 BEEC subjects. Seven subjects had a synonymous SNP p.Leu264Leu (rs17850223). No additional SNPs were present in the coding region of *OTX1*.

## Discussion

During the past several years many microdeletion syndromes have been identified using microarray methods [40]. This is especially true for subjects with intellectual disability who commonly have other abnormalities, such as GU defects. We identified seven male subjects, six with GU defects involving the genitalia, who share a commonly deleted region in 2p15 involving only *OTX1* (Fig.1). The 2p15p16.1 microdeletion syndrome was described in ten subjects focusing mainly on their neurodevelopmental phenotypes without emphasizing that seven of them also have identifiable GU defects, predominantly affecting the testes and kidneys. The first report of the 2p15p16.1 microdeletion was in two individuals: a boy with small testes and penis, hydronephrosis, and VUR and a girl with a multicystic kidney and hydronephosis [28]. To date, three of six female patients reported with 2p15p16.1 microdeletions (not including *OTX1*) have GU defects, one presenting with hypogonadism and two with hydronephrosis [22], [24], [26]–[28]. All four male patients with 2p15p16.1 microdeletions (only one of which includes *OTX1*) have GU defects including testicular (100%), kidney (50%), and penile (25%) defects [21], [23], [28], [29] (Fig. 1). Four genes are present in the minimally deleted region of these patients. *AHSA2* and *SNOR70B* have unknown functions. The other two, *USP34* and *XPO1*, are better characterized, but no role in GU development is attributed to them. USP34 functions downstream of the β-catenin complex to control the stability of axin as well as enhance NF-κB activation [41], [42]. *XPO1* is over-expressed 2–4 fold in cancer [43]. More recently, a second 2p14p15 microdeletion syndrome was described in three subjects. The first description is of a boy and a girl with mild intellectual disability and no GU defects with *OTX1* deleted only in the girl [30]. In an additional case, *OTX1* is deleted in a boy with intellectual disability and cryptorchidism [31]. A small deletion in subject-1 (encompassing only *OTX1*) and the minimal overlapping region between subjects-2 and -3 (which includes *EHBP1, OTX1* and *WDPCP*), allows us to suggest that *OTX1* is involved in normal genitourinary development.

We cannot exclude the possibility that the two additional genes deleted in the minimal region of subjects 2–7, *EHBP1* and *WDPCP*, could also be implicated in the diverse GU phenotypic defects observed. This is particularly true for *WDPCP* since mutations may be associated with Bardet-Biedl syndrome 15 (BBS15) [44]. BBS15 is characterized by rod-cone dystrophy, truncal obesity, postaxial polydactyly, cognitive impairment, hypogonadism, cryptorchidism, micropenis, and renal abnormalities in which renal disease is a major cause of morbidity and mortality. On the other hand, *EHBP1* is a putative genetic susceptibility loci for prostate cancer with no association with GU defects [45]. In our cohort subjects 2–7 exhibit larger deletions involving additional genes that may contribute to their GU and other phenotypic anomalies.

OTX1 and OTX2 are transcription factors with important roles in controlling specification, maintenance, and organ regionalization. The Gudmap database indicates that both *OTX* genes are expressed in mouse ureter, testis, and ovary in Theiler stage 23 [46], [47]. In the prepubescent stage, *Otx1-*deficient mice have seizures along with growth retardation and gonadal defects attributed to low levels of pituitary hormones (growth hormone, FSH, and LH), which dramatically affect ovary and testis size and architecture. Nevertheless, *Otx1*'s role in modulation of pituitary hormones is transient, and four-month-old mice show normal hormonal levels and gonadal size [33]. *Otx2* null embryos die embryonically because of major body abnormalities, including absence of the neuroectoderm [48]. However, *Otx2* heterozygous male mice display compromised fertility (reduced LH levels and testicular weight) due to a defect in the development, number, and migration of GnRH neurons [49].

OTXs are important in cell fate differentiation, and a specific threshold of OTX proteins is required for proper SHH signaling [50]. The SHH signaling pathway coordinates the formation of the bladder, internal urethra, and genitalia [51]. Heterozygous *Otx1* mice are not fully characterized, but correct dosage of Otx2 is critical for normal fertility and testis size. Otx1 and Otx2 have functional similarity and interchangeable roles [52]. *OTX2* could compensate for *OTX1* deficiency in levels that vary among subjects. Since *OTX1* haploinsufficiency could have a direct effect on the SHH signaling pathway, which is crucial for development of the bladder and genitalia, this may explain the range of GU defects seen in our patients and the bladder phenotype present in subject-1. Six of our subjects and two previously reported cases have genital defects including cryptorchidism, hypogonadism, micropenis, epispadias, and foreskin and scrotal anomalies. There is no mention of abnormal testicular descent in *Otx1-*null mice, but in our experience, unless the testes are beside the kidneys (abdominal cryptorchidism), an abnormal testis position is often overlooked and lesser degrees of cryptorchidism just above the inguinal ring are not reported.

Regardless, we cannot exclude the possibility that the hypogonadism and cryptorchidism present in some subjects may be secondary to a pituitary defect similar to that occurring in the *Otx1* null mice [33]. Fetal defects in the pituitary-Leydig cell axis are associated with cryptorchidism [53]. The hypothalamic-pituitary axis regulates testicular hormone secretion in the second half of fetal life and FSH controls the Sertoli cell proliferation responsible for testis volume increase upon the onset of spermatogenesis. LH regulates the Leydig cell androgen and INSL3 secretion required for testis growth and descent [54]. Hypogonadism resulting from *OTX1* haploinsuficiency could lead to micropenis and cryptorchidism in these patients. In addition, *Otx1* null mice suffer from generalized seizures and growth retardation [32]. In concordance with these mouse findings, two of our subjects, as well as two additional cases reported in the literature, have seizures and two have short stature (although this did not correlate with age in our cohort).

One caveat of our study is that two of the seven pregnancies were complicated by maternal hypertension (Subject-1 and -3) and another by progesterone supplementation for the first 11 weeks of pregnancy (Subject-2). These additional conditions/medical interventions may have impacted normal GU development. In addition to genetic factors, placental insufficiency, low birth weight, and twinning are implicated as contributing to the etiology of cryptorchidism [55], [56]. The impact of preeclampsia, in vitro fertilization, and exposure to endocrine disrupters on normal GU development is controversial and the results vary between studies [55], [56]. With respect to BEEC, preconception or first trimester exposure to alcohol, environmental toxins, or maternal disease are not associated with the anomaly [57]. A direct relationship between maternal hypertension and progesterone intake is not obvious in boys born with cryptorchidism or BEEC; accordingly, OTX1 microdeletion remains a strong candidate genomic condition associated with the GU defect observed in these patients.

The deleted region shared by our subjects is between the minimum deletion region of the 2p15p16.1 [28] and 2p14p15 microdeletion syndromes [31]. The presence of GU defects in 86% of our cohort, in 70% of subjects with 2p15p16.1 deletions, and in 33% of the subjects with 2p14p15 deletions suggests that this region contains genes important in GU development. Our results may associate deletions of *OTX1* with GU anomalies, possibly through alterations of the SHH signaling pathway. Larger deletions in many of these subjects (including multiple genes) result in additional features, such as developmental delay, intellectual disability, and dysmorphic features. Of note, *OTX1* may not be the only gene involved in GU development in this region since three patients with 2p15p16.1 deletions that do not include *OTX1* also display GU defects. *OTX1* deletion could affect neighboring genes, in particular the gene *WDPCP* in which mutations were associated with hypogonadism, cryptorchidism, micropenis, and renal abnormalities [44]. *OTX1* microdeletions are rare in populations clinically tested by aCGH, varying from 0.010–0.019% in BCM and SG clinical laboratories, respectively. In addition, the 85 normal controls tested in our laboratory and the 8329 controls analyzed by Cooper et al. [58] did not exhibit CNVs in *OTX1*. The ISCA database includes three individuals with CNVs containing *OTX1*, but only one individual has a microdeletion (5.3 Mb). Decipher has four subjects with microdeletions ranging from 0.51–3.21 Mb. Two of them are boys with no phenotype recorded and two are girls with no GU defects reported. In addition, Decipher lists seven subjects with microduplications. Four have large duplications (15.32–89.01 Mb) and no reported GU defects. The other three have small duplications (0.72–4.66 Mb) including a female (0.72 Mb) with an enlarged kidney, a male with cryptorchidism (4.66 Mb), and a third male with no reported phenotype. The absence of controls with CNVs in *OTX1* and the phenotypes present in patients with microdeletions suggests the importance of proper dosage of *OTX1* and 2p15 genes in GU tract development. Understanding the molecular mechanisms behind the pathogenesis of GU defects is important for genetic counseling and the implementation of therapeutic interventions. The present study suggests that chromosomal region 2p15 and *OTX1* are involved in GU tract development, but further detailed studies are needed to identify a causal relationship.
